# Diffuse anorectal melanoma; review of the current diagnostic and treatment aspects based on a case report

**DOI:** 10.1186/1477-7819-7-64

**Published:** 2009-08-11

**Authors:** Christos N Stoidis, Basileios G Spyropoulos, Evangelos P Misiakos, Christos K Fountzilas, Panorea P Paraskeva, Constantine I Fotiadis

**Affiliations:** 1 3rd Department of Surgery, University of Athens Medical School, Attikon University Hospital, Athens, Greece; 2 Department of Internal Medicine, Athens Navy Hospital, Athens, Greece; 3 2nd Department of Propedeutic Surgery, University of Athens Medical School, Laikon General Hospital, Athens, Greece

## Abstract

Primary anorectal melanoma is a rare and aggressive disease. Patients commonly complain for changes in bowel habits and rectal bleeding, and proctoscopically they mostly appear as non pigmented or lightly pigmented polypoid lesions. Such a lesion should always raise a high index of suspicion in any gastroenterologist or surgeon to prompt surgery, since early radical excision is the only treatment option.

Herein, we report a case of a 57-year-old man with a diffuse anal canal melanoma and give reference to the current diagnostic and treatment options.

## Introduction

Malignant melanoma of the anal canal accounts for 1%–3% of all anal canal tumors, yet it is an uncommon site of primary melanoma with almost equal male to female ratio and with an average age of presentation between the fifth and the sixth decades of life [1]. It is documented that it is the third most common site following the skin and the eye, while the majority of patients in the world literature are Caucasian [2]. The lesions can be located in the anal canal, rectum or both with the majority of them arising from the dentate line of the anal canal. They tend to spread submucosally, and by the time they cause symptoms the extent of invasion is usually beyond surgical cure.

The etiology of the disease is unknown. A history of sun exposure is not likely to have had an impact on their development, while recent epidemiologic data indicate a bimodal age distribution [3]. Currently, there is little information whether an infection with the human papilloma virus plays a role in the tumorigenesis of anorectal melanoma.

Prompt surgery seems to be the only treatment option since current chemotherapy and radiotherapy alone have been proved ineffective. The development of novel therapies to treat malignant melanoma will hopefully improve the clinical outcome.

## Case report

A 57-year-old male patient was admitted to our department with a chief complaint of intermittent rectal bleeding and constipation that had started 6 months ago. All physical findings were undiagnostic except digital examination which revealed a wide-based, fixed, ulcerative mass 2,8 cm above the anal sphincter, just behind the anal verge, without evidence of invasion in the sphincter, growth outside the rectum or enlarged lymph nodes. Laboratory tests disclosed red blood cell count: 3,8 × 105/mm^3^, hemoglobin: 8,3 g/dl, and hematocrit: 27,4%. Proctosigmoidoscopy confirmed digital findings and included the anorectal melanoma in our differential diagnosis. Nevertheless, despite the characteristic nature of the tumor multiple biopsies were taken in order to establish the diagnosis. The serum level of 5-S-cysteinyldopa (5-S-CD) was elevated at 60 nmol/l (normal range, 1.5–8.0 nmol/l).

Histopathologically, the tumor consisted of spindle-shaped cells, resembling fibrosarcoma cells, with melanin pigment. The tumor cells had invaded the muscularis propria of the rectum, and lymphatic invasion was noted. Three perirectal nodes contained histologically evident metastasis. Thus, the pathologic stage was III Á according to the AJCC TNM classification (AJCC Cancer Staging Manual, 6th edition-2002). After surgery, the serum 5-S-CD level decreased to 5.8 nmol/l.

**Figure 1:**
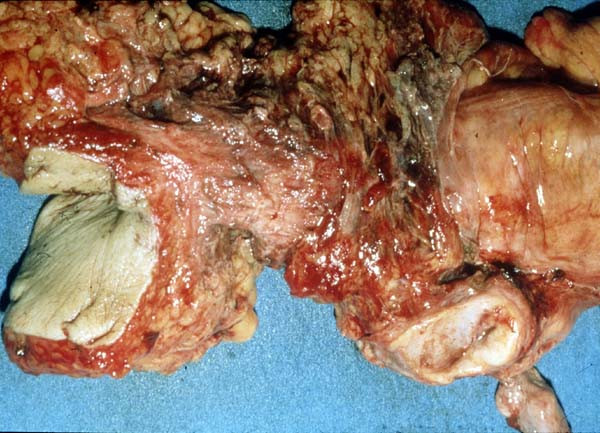
**The excised specimen of our case**.

**Figure 2:**
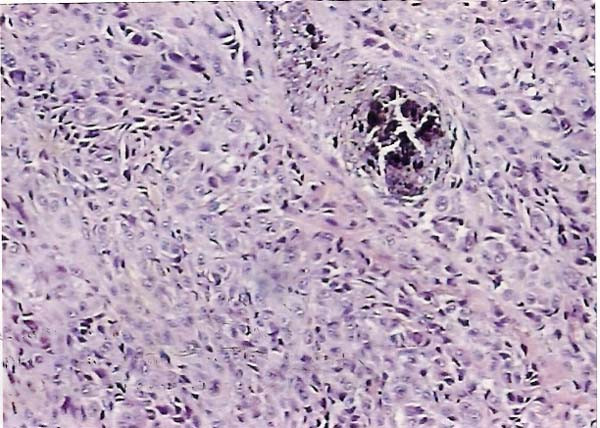
**Microscopic view of the permanent section. Spindle-shaped cells, resembling fibrosarcoma cells, with melanin pigment**.

During the immunohistochemical control the S-100 protein stain was strongly positive, the HMB-45 stain positive, the N-Cam stain focally positive in few isolated cells and negative were the stains for Mart-1 Tyrosynase, Leu7, Chromogranin, Synaptorysin, CD117, CD34, Actin, Desmin, EMA, HMWK, LMWK, Ker 8–18, Ker 20 and LCA. The differential diagnosis included neuroendocrine neoplasm, gastrointestinal stromal tumor (GIST), paragangglioma and neoplasm of melanocytic origin. The positive expression of the HMB-45 and S-100 protein antibodies set the diagnosis of the malignant neoplasm of melanocytic origin (malignant melanoma) as the most prevalent. CT scans proved no metastatic disease and serum levels of tumor markers including carcinoembryonic antigen and Ca 19-9 were within normal ranges. The diagnosis of anal canal melanoma was based on the proctoscopic biopsies. An endoluminal ultrasound proved a 10 mm tumor thickness. A typical abdominoperineal resection (APR) was performed with extent resection of the bilateral iguinal, pelvic sidewall and mesorectal lymph nodes (Figure 1, 2). The primary treatment was completed with adjuvant region radiation therapy. The patient recovered uneventfully. Six months later during the first follow up examination, multiple metastatic lesions were recognized. The relapse site was the liver. The patient submitted to chemotherapy and passed away 20 months later from disseminated disease.

Several questions arose after this aggressive recrudescence of the disease. Is really anorectal melanoma an incurable disease and which should be the best surgical treatment for it? Finally, in what way do the prognostic and clinicopathological factors influence the outcome of this disease?

## Discussion

As with other anal canal tumors there is often a delay in diagnosis that results in advanced stage disease at the time of presentation. Because anorectal melanomas are rare, staging of the disease has previously been limited to local, regional, and distant disease [4]. Most patients with such melanomas complain for bleeding, pain, or an anal mass. Digital examination provides information concerning size, fixation and ulceration of the tumor and proctosigmoidoscopy may be suggestive of anorectal melanoma when pigmentation is obvious. Anal canal melanomas present no specific clinical manifestations and due to their polypoid nature they are often misdiagnosed as a thrombosed hemorrhoid [5]. Obviously, the inexperienced endoscopist should always have this rare pathology in mind in order to avoid misdiagnosing this lesion as a thrombosed hemorrhoid.

Endoluminal ultrasound is an established mode of evaluation of the tumor thickness and its' nodal status, but the diagnosis must always be based on the permanent sections due tendency of amelanotic types to masquerade as lymphoma, sarcoma or undifferentiated carcinoma [6]. Immunohistochemistry may also be helpful in the diagnosis of anorectal melanomas; S 100 protein, HMB-45, Melan A, and MiTF (microphthalmia-transcription-factor) are useful immunohistochemical markers.

Metastases occur via lymphatic and hematogenous routes and it has been reported that 38% of patients have already metastatic disease at the time of diagnosis [7]. Lymphatic spread to mesenteric nodes is more common than to inguinal nodes while lungs, liver and bones are the most frequent sites of distant metastases.

In the absence of any metastasis surgical therapy is indicated. Most series report no difference in survival in patients treated by wide local excision or abdominoperineal resection (APR) although the latter has proved more effective to control the local disease but, without clear improvement in survival [8,9]. This is caused by the fact that most recurrences occur systemically regardless of the initial surgical procedure. However, a recent study suggests that sphincter-sparing local excision and adjuvant radiation is well tolerated and can effectively control local-regional disease while avoiding the functional morbidity of APR [10]. The benefits of LE are clear and include quicker recovery from a less invasive procedure, minimal impact on bowel function, and no need for a stoma. Prophylactic lymph node resection is of no value whereas therapeutic lymph node dissection is indicated only in the presence of positive inguinal nodes.

Introducing sentinel lymph node mapping (SLNM) using different radioactive tracers and endoscopic ultrasonography in recent years has influenced the extent of surgical resection. Few case reports on the use of SLNM in anal melanoma have been reported [11]. Although SLNM and biopsy in anal melanoma has not yet become a standard of care, it is technically feasible as reported in these case reports. SLNM seems to be helpful in preventing understaging patients who are pathologically node-positive but clinically node-negative. Long-term follow-up of the impact of the possible finding of micrometastases is needed.

In rare cases of anorectal melanomas which tend to block the anal orifice, palliative surgical treatment is indicated [12]. In severely ill patients unable to tolerate any surgical procedure, intramural injections of natural interferon-beta and systemic administration of dacarbazine has been proposed with good results [13]. No systemic therapy regimen for metastatic anal melanoma is considered standard of care. Treatment is based on drugs developed for advanced cutaneous melanoma and includes cisplatin, vinblastine, dacarbazine, INF, and interleukin-2, although given the clinical, biologic, and molecular differences, mucosal and cutaneous melanomas may be distinct disease entities [14]. After temozolomide has shown efficacy comparable to dacarbazine in a randomized trial of cutaneous melanoma, a combination of temozolomide, cisplatin, and liposomal doxorubicin in one patient with metastatic anal melanoma was used with encouraging results [15].

The presence of perineural invasion (PNI) is an important prognostic factor and should be considered in future clinical trials [16]. Notably, tumor thickness seems to be a strong predictive factor for the risk of local recurrences. Anorectal melanoma seems to be similar to cutaneous melanoma, for which tumor thickness is used to plan therapeutic procedures. Hence, for anorectal melanoma tumor thickness may also be used as a guideline, i.e. in early disease with a tumor thickness below 1 mm a local sphincter-saving excision with a 1-cm safety margin and in cases of tumor thickness between 1 and 4 mm a local sphincter-saving excision with a safety margin of 2 cm seems to be adequate [17]. Tumors with thickness above 4 mm should be treated with APR to avoid local complications; even so, there will be a stoma and the risk of urinary and sexual dysfunction [18].

Nevertheless, despite sporadic promising reports, regardless of surgical approach, anorectal melanoma remains a highly lethal malignancy with overall 5-years survival rate less than 20% according to all reported series [19].

Regarding our case, there has been a long debate regarding the extent of resection, which was necessary to optimally treat the melanoma. APR procedure, based on the clinicopathological features of the tumor was preferred, although the benefits of LE are clear. The need for regional lymphadenectomy has been at the center of the debate. During APR, mesorectal lymph nodes are resected en bloc with the primary tumor. Although the patient's outcome was poor, we were able to identify risk factors associated with survival and prognosis. Clinical symptoms, PNI, tumor thickness and diameter, spindle cell histology, mural involvement and necrosis may ultimately impact outcome. Finally, we hypothesized that systemic dissemination is an early event in tumorigenesis and by the time the lesion is clinically apparent, micrometastases are well established.

## Conclusion

A standard approach to managing this aggressive tumor has not been established because of the limited number of patients and the retrospective design of all anal melanoma reports. Advanced anorectal melanoma most likely represents a systemic disease at time of diagnosis. Therefore, therapy of the primary tumor has no influence on the systemic course of the disease. This is not necessarily true for early disease.

For anal melanomas, as for any melanoma, the biological control of the disease is crucial. It is highly desirable that the new modality of medical, biological or immunological therapies will improve the final outcome of these patients. So far, for this rare tumor, a surgical procedure which can achieve a complete local excision with the highest respect of the functional aspects and quality of life remains the best therapeutic approach to be applied.

Hence, therapeutic strategies should be adjusted to the prognosis of the disease. Unfortunately, prognostic parameters for anorectal melanoma remain to be defined. Only a few studies addressed this pertinent question.

The aim of this report is to emphasize that early diagnosis is the key to improved survival rate for patients with these unusual variants of melanoma.

## Consent

Written informed consent was obtained from the patient for publication of this case report and accompanying images. A copy of the written consent is available for review by the Editor-in-Chief of this journal.

## Competing interests

The authors declare that they have no competing interests.

## Authors' contributions

CIF was the patient's surgeons and has been involved in drafting the manuscript and revising it critically for important intellectual content. EPM, CNS, BGS, PPP and CKF have made contributions to conception and design. CNS contributed to the analysis and interpretation of data. All authors read and approved the final manuscript. All authors contributed equally to the final draft of the manuscript. CIF has given the final approval of the version to be published.
